# Tonsil fibromatosis

**DOI:** 10.1016/S1808-8694(15)30617-0

**Published:** 2015-10-18

**Authors:** Juliana Sato, Antonio Augusto de Lima Pontes, Ricardo Frazatto, Reginaldo Raimundo Fujita

**Affiliations:** 1 ENT, Fellow in pediatric otorhinolaryngology at UNIFESP-EPM.; 2 MD, third-year specialization student at Unifesp-EPM.; 3 MSc in otorhinolaryngology and head and neck surgery at UNIFESP-EPM, ENT.; 4 Adjunct Professor at the Department of Otorhinolaryngology and Head and Neck Surgery at UNIFESP-EPM, Head of the Pediatric ENT Clinic at UNIFESP-EPM. Universidade Federal de São Paulo - Escola Paulista de Medicina

**Keywords:** tonsil, fibromatosis

## INTRODUCTION

Fibromatosis is a non-metastatic, however locally invasive tumor that may infiltrate muscle, fat, and bone. It may be fatal depending on its location. Tumors in the head and neck are particularly dangerous^1^.

These fibroblast tumors have poorly defined margins and are not encapsulated^2^. Anaplasia and invasion of nerves and vessels are not present; mitosis is rare and there may be collagen between tumor cells^1^.

This paper aims to present a case of fibromatosis involving a child's tonsils and discuss issues related to treatment and prognosis.

## CASE REPORT

The patient is a 10-year-old male, brown, born in Maceió. In 2002, he came to our service with a history of snoring, mouth breathing, and repetition tonsillitis. He underwent an adenoidectomy at the age of four. In the examination he was seen to have grade IV tonsil hypertrophy and reduced air column in the rhinopharynx as seen in the radiography of the cavum, being thus referred to an adenotonsillectomy.

**Figure 1:**
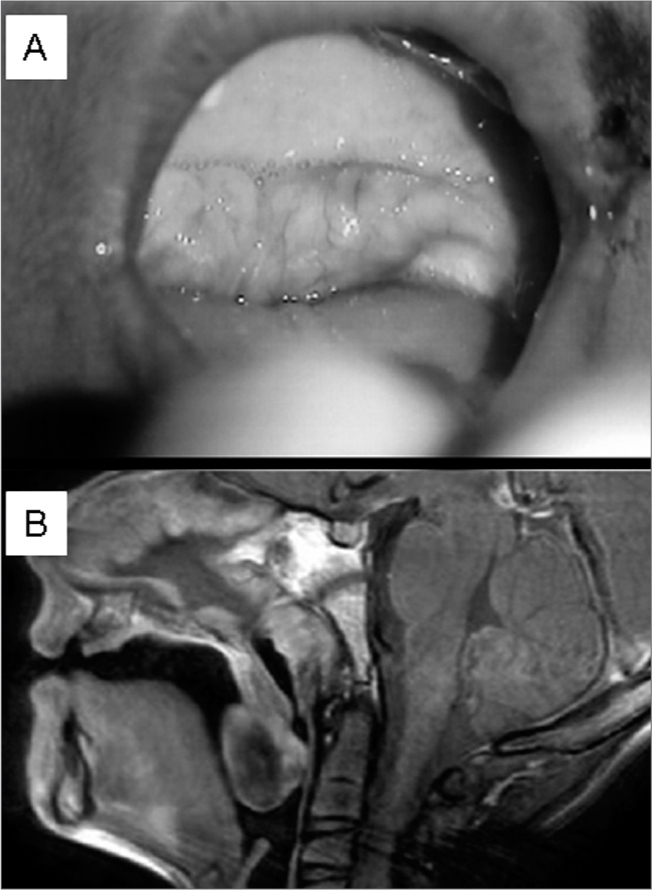
Tumor invading the soft palate and tonsillar pillars.

During surgery it was verified that his tonsils were unusually hard, and adhesions between the tongue base and the uvula were observed. The adenotonsillectomy was performed and tonsil pathology tests were positive for a fibroblastic proliferation consistent with fibromatosis.

A year later the patient began to lose weight and experience difficulty swallowing solid foods. Oroscopy revealed a tumor invading the soft palate and tonsillar pillars (Figure). Laryngoscopy did not show any involvement of the pharyngeal posterior wall. The MRI revealed a solid, well-limited lesion involving distal portions of the soft palate, measuring 3.0x2.5x2.5cm, with marginal enhancement after contrast uptake. Hard palate, tongue base, posterior pharyngeal wall, and skull base were not involved. He was referred to surgical resection of the lesion with a safety margin of 2.0 cm.

## DISCUSSION

Fibromatosis occurs in the head and neck in 10-35% of the cases1. Stout was the first to identify and describe the disease's aggressive manifestation in 1954^3^. This disease affects predominantly children and young adults, and develops slowly in about half the patients^2^.

Resection with broad safety margins is the treatment of choice, a challenging goal for head and neck tumor removal without leaving any sequelae^1^, ^2^, ^4^. In adults, the recommended safety margin is three centimeters^4^. Post-surgery relapse is seen in 23.8% to 57% of the patients ^5^. Some authors indicate radical clearance for patients with neck fibromatosis^1^.

Fowler^5^ studied 31 cases of fibromatosis in the oral cavity, and found that the cheeks, tongue, and submaxillary region were the most commonly affected sites.

There is no consensus as to the use of chemo or radiotherapy, but both have been used as adjuvant treatment in inoperable tumors. One of the downsides of radiotherapy is the high dosage required to treat the tumor, as it may damage bone epiphysis and thus impair the patient's growth^2^, ^4^.

Chemotherapy alone does not seem to be a curative treatment, but may be effective in managing inoperable tumors and in reducing the size of the tumor before surgery. Some studies indicate that tumor growth may be impacted by estrogen, and that adjuvant therapy with tamoxifen might be useful. Others looked into non-steroidal anti-inflammatory drugs due to their impact in prostaglandin metabolization^4^.

In the literature we found only one case of tonsil fibromatosis, also in a child, with invasion of the pterygopalatine fossa. Cure was only possible through broad tumor resection^6^.

## CONCLUSION

This paper illustrates the difficulties in treating a rare condition as head and neck fibromatosis in children. Despite the various treatments proposed for the disease, not all can be offered to children, thus turning it into a benign disease of challenging management.
